# Editorial: Biomarkers of response to interventions in psychiatry

**DOI:** 10.3389/fpsyt.2026.1770939

**Published:** 2026-01-22

**Authors:** Sara B. Franco, Kevin Pacheco-Barrios, Ricardo R. Uchida, Lucas Murrins Marques

**Affiliations:** 1 Principles and Practice of Clinical Research Program, Executive and Continuing Professional Education (ECPE), Harvard T.H. ChanSchool of Public Health, Boston, MA, United States; 2 Neuromodulation Center and Center for Clinical Research Learning, Spaulding Rehabilitation Hospital and Massachusetts General Hospital, Harvard Medical School, Boston, MA, United States; 3 Universidad San Ignacio de Loyola, Vicerrectorado de Investigación, Unidad de Investigación para la Generación y Síntesis de Evidencias en Salud, Lima, Peru; 4 Mental Health Department, Santa Casa de São Paulo School of Medical Sciences, São Paulo, SP, Brazil; 5 Artificial Intelligence Research Division, Infinity Doctors Inc., Miami, FL, United States; 6 Eco-Wellbeing & Affective Health Laboratory (EWAH Lab), São Paulo, SP, Brazil

**Keywords:** biomarkers, mental health, precision psychiatry medicine, psychiatric interventions, translational research, treatment response

Mental disorders remain among the leading causes of disability and premature mortality worldwide, with a growing burden across both high-income and low- and middle-income countries. Despite substantial advances in pharmacological and psychosocial treatments, clinical decision-making in psychiatry continues to rely heavily on subjective symptom reports and trial-and-error approaches. This limitation has fueled increasing interest in biomarkers capable of objectively capturing biological changes associated with treatment response, remission, or resistance. Against this background, the Research Topic Biomarkers of Response to Interventions in Psychiatry was conceived to advance the field beyond biomarker discovery for diagnostic classification and toward their practical application in monitoring therapeutic outcomes.

A central motivation of this Topic was to address a persistent gap in the literature: while numerous studies focus on identifying biomarkers associated with psychiatric diagnoses, fewer investigate how biological measures change dynamically in response to interventions or how they can inform treatment optimization in real-world clinical contexts. The six contributions included in this Research Topic collectively illustrate the breadth of this emerging field, spanning neuropsychological, molecular, immunological, and neurophysiological biomarkers, as well as diverse psychiatric populations and intervention settings.

Several contributions highlight the potential of biological markers to predict or track response to antidepressant treatment. Kim et al. examined employment-dependent associations between serum biomarkers and short- and long-term antidepressant outcomes, underscoring the importance of contextual and psychosocial factors in shaping biological response profiles. Their findings reinforce the notion that biomarkers do not operate in isolation but interact with social determinants of health, a critical consideration for precision psychiatry. Complementing this perspective, Targum et al. demonstrated that alterations in the heterotrimeric G protein Gαs may predict antidepressant response in individuals with major depressive disorder, offering insights into intracellular signaling mechanisms underlying treatment efficacy.

The integration of peripheral biomarkers was further explored in studies examining inflammatory and hematological markers. Zhou et al. reported a U-shaped association between myeloperoxidase levels and anxiety risk in a large Chinese population, highlighting the complexity of immune-related biomarkers and the potential pitfalls of assuming linear associations between biological markers and psychiatric outcomes. In a related vein, Dai et al. investigated hematological, biochemical, and cytokine profiles in hospitalized psychiatric patients with COVID-19, illustrating how systemic inflammatory processes may interact with psychiatric vulnerability and treatment trajectories in medically complex populations.

Beyond blood-based biomarkers, this Research Topic also emphasizes neurophysiological and neurobehavioral approaches to treatment monitoring. de Deus et al. presented a detailed study protocol exploring electroretinography as a biomarker for predicting and monitoring antidepressant response in major depressive disorder. This work exemplifies the growing interest in retinal and sensory biomarkers as accessible proxies for central nervous system functioning. Similarly, Liu et al. demonstrated the utility of the tree drawing test as an auxiliary tool for evaluating treatment outcomes in schizophrenia, highlighting the continued relevance of integrative approaches that bridge neuropsychological assessment and biological inference.

Taken together, these contributions underscore several cross-cutting themes. First, biomarkers of treatment response in psychiatry are inherently multidimensional, encompassing molecular, physiological, neurophysiological, and behavioral levels of analysis. Second, the clinical utility of biomarkers depends not only on their biological validity but also on their feasibility, interpretability, and integration into routine care. Third, contextual factors—such as employment status, medical comorbidities, and broader social conditions—play a critical role in shaping biomarker–treatment relationships and must be explicitly considered in future research.

Importantly, the studies included in this Research Topic move the field closer to actionable precision psychiatry. Rather than framing biomarkers solely as static indicators of disease state, they emphasize dynamic biological processes that unfold over the course of treatment. This shift has significant implications for clinical practice, including the potential for early identification of non-responders, individualized treatment adjustments, and improved long-term outcomes.

In conclusion, Biomarkers of Response to Interventions in Psychiatry provides a timely and integrative snapshot of current efforts to translate biological insights into meaningful clinical applications. By showcasing diverse methodologies and clinical contexts, this Research Topic highlights both the promise and the challenges of biomarker-guided interventions in psychiatry. Future work will benefit from longitudinal designs, multimodal integration, and closer alignment between biological measures and patient-centered outcomes. Ultimately, advancing the science of treatment-response biomarkers holds the potential to transform psychiatric care from reactive symptom management to proactive, personalized intervention strategies.

